# Interaction of inhibition and synaptic plasticity in a model of the hippocampal CA1 microcircuit

**DOI:** 10.1186/1471-2202-12-S1-P126

**Published:** 2011-07-18

**Authors:** Aušra Saudargienė, Giedrė Slivko, Bruce P Graham

**Affiliations:** 1 Department of Informatics, Vytautas Magnus University, Kaunas, LT-44248, Lithuania; 2 Institute of Computer Science and Mathematics, University of Stirling, Stirling FK9 4LA, UK

## 

Cellular activity in the CA1 area of the hippocampus waxes and wanes at theta frequency (4-7Hz) when a rat is exploring an environment. Perisomatic inhibition onto pyramidal cells from basket cells and axoaxonic cells tends to be strongest out of phase with pyramidal cell activity, in a so-called storage cycle, whereas dendritic inhibition, mediated by bistratified and oriens lacunosum-moleculare (OLM) cells is strongest in phase with pyramidal cell activity [1], in a so-called recall cycle. Synaptic plasticity also varies across the theta cycle, from strong LTP to LTD, putatively corresponding to the storage and recall cycles for information patterns encoded in pyramidal cell activity [2]. The mechanisms underpinning the phasic changes in plasticity are not clear, but it is likely that inhibition plays a role by affecting levels of electrical activity and calcium levels at synapses. Calcium levels at dendritic synapses could reach the amplitudes required for LTP when inhibition is restricted to the perisomatic region, but may be restricted to amplitudes that result in LTD when inhibition is strong in the dendrites.

We explore this hypothesis using a detailed multicompartmental model of a CA1 pyramidal cell [3] which is driven by spatially-focused patterns of excitation and inhibition. We employ a model of the CA1 microcircuit [4] that also includes basket, axoaxonic, bistratified and OLM cells and inputs from the entorhinal cortex (EC), the CA3 Schaffer collaterals and medial septum. A trigger of synaptic modifications, the intracellular calcium concentration, is modeled in a spine that expresses AMPA/NMDA-receptor-mediated synaptic responses and is located on a stratum radiatum (SR) dendrite of the CA1 pyramidal cell. The resulting calcium signals are used in a model of spike-timing-dependent synaptic plasticity [5] to predict the synaptic modifications across the theta cycle.

We show that in the storage cycle, EC input-induced dendritic spikes propagate from the stratum lacunosum moleculare (SLM) dendrites to the SR dendritic regions in the CA1 pyramidal cell, coincide with the CA3 inputs, induce large calcium influx into the spine on the SR dendrite and cause LTP [6]. In the recall cycle, dendritic inhibition provided by bistratified and OLM cells prevents dendritic spike propagation into the SR region, restricts calcium increase and results in LTD induction. Removing dendritic inhibition in recall cycles allows dendritic spikes reaching SR dendrites and reverts LTD to LTP.

The results suggest that dendritic inhibition acts as a switch that prevents LTP and promotes LTD during the recall memories and in forgetting no longer relevant memories.
